# Sharp margin and geographic shape: systematic evaluation of two novel CT features in COVID-19 pneumonia

**DOI:** 10.1259/bjro.20200026

**Published:** 2020-07-08

**Authors:** Jan Schaible, Stefanie Meiler, Florian Poschenrieder, Gregor Scharf, Florian Zeman, Janine Rennert, Benedikt Pregler, Charlotte Knobloch, Henning Kleine, Sina Grote, Christian Stroszczynski, Niels Zorger, Okka Wilkea Hamer

**Affiliations:** 1 Department of Radiology, Regensburg University Medical Center, Franz-Josef-Strauss-Allee 11, 93053 , Regensburg, Germany; 2 Center for Clinical Studies, Regensburg University Medical Center, Franz-Josef-Strauss-Allee 11, 93053, Regensburg, Germany; 3 Department of Pneumology, Hospital Barmherige Brüder, Prüfeninger Strasse 86 93049, Regensburg, Germany; 4 Department of Radiology, Hospital Barmherige Brüder, Prüfeninger Strasse 86 93049, Regensburg, Germany

## Abstract

**Objective:**

CT is important in the care of patients with COVID-19 pneumonia. However, specificity might be poor in the absence of a clinical and epidemiological context. The goal of this work was to systematically evaluate two novel CT features (sharp margin and geographic shape) of COVID-19 pneumonia.

**Methods:**

All patients with reverse transcription polymerase chain reaction proven COVID-19 pneumonia and chest CT between March first and April 15, 2020 were retrospectively identified from two tertiary care hospitals in Germany. The CTs were evaluated regarding the presence of typical CT signs (*e.g.* ground glass opacitiy, consolidation, crazy paving). Moreover, the shape of the opacifications (round, geographic, curvilinear) and their margin (unsharp, sharp) was determined.

**Results:**

The study population comprised 108 patients (64 male) with a mean age of 59.6 years. Ground glass opacities (96%) and consolidation (75%) were the most prevalent CT signs. Crazy paving was seen in 17%, bronchial dilatation in 21%, air bronchogram in 29%, vessel enlargement in 47%, cavitation in 0%, lymphadenopathy in 32%, pleural effusion in 16%. Round configuration of densities was present in 41% of CTs, geographic shape in 27% and curvilinear opacities in 44%. 79% of opacifications were at least partially sharply marginated. In almost all cases, the lung was affected bilaterally (94%).

**Conclusion:**

The CT pattern of COVID-19 pneumonia in a cohort from Germany was in accordance with prior studies. However, we identified two novel CT signs of COVID-19 pneumonia which have so far not been systematically evaluated. A sharp border and geographic shape of opacifications were frequently observed.

**Advances in knowledge:**

The newly described CT features “sharp margin” and “geographic shape” of opacifications in patients with COVID-19 pneumonia might help to increase specificity of CT.

## Introduction

Since the outbreak of coronavirus disease 2019 (COVID-19) in December 2019, several studies reporting the morphology of COVID-19 pneumonia in chest CT have been published.^1–9^ Unlike most other viral pneumonias CT morphology of COVID-19 pneumonia seems to exhibit remarkably similar features in many patients in particular in the acute phase. This circumstance has triggered the development of assessment and reporting schemes categorizing the findings in different groups of likelihood for COVID-19 pneumonia.^10,11^ Nevertheless, specificity of CT for COVID-19 pneumonia is reported to be variable with reported numbers as low as 25%.^12^ Hence, identification of CT features which occur frequently in patients with COVID-19 pneumonia and which allow discrimination of COVID-19 pneumonia from differential diagnoses is mandatory. According to the review of images published in literature and our own experience, we identified two CT features of COVID-19 pneumonia that to the best of our knowledge have so far not been systematically evaluated. The opacifiations in COVID-19 pneumonia seem often to be sharply demarcated with respect to the surrounding healthy lung tissue. When multiple adjacent pulmonary lobules are involved the shape of the opacification becomes geographic. This morphology is otherwise not often seen in chest CT and would be strongly suggestive for COVID-19 pneumonia during the pandemic.

The aim of this study was to evaluate CT morphology of reverse transcription polymerase chain reaction (RT-PCR) proven COVID-19 pneumonia in a German cohort with special emphasis on the frequency of the empirically observed but so far not systematically evaluated novel CT features.

## Methods and materials

### Patient population

This retrospective study was approved by the institutional ethics committee. Written informed consent was waived. All procedures performed in studies involving human participants were in accordance with the ethical standards of the institutional and/or national research committee and with the 1964 Helsinki declaration and its later amendments or comparable ethical standards.

The inclusion criteria were consecutive adult patients (≥18 years old) with RT-PCR positive for SARS-CoV-2 and a chest CT performed between March 1 and April 15, 2020. In case of more than one CT per patient, the first CT was included into the analysis. The PCR was obtained from a throat swab in 35 cases, from tracheal secretion or sputum in 61 cases and from bronchoalveolar lavage in 12 cases. Exclusion criteria were a non-diagnostic CT, *e.g.* due to motion-artifacts. Patients were identified by means of a full-text database query of all CT-scans performed between March 1 and April 15, 2020 using the term “*COVID*” OR *SARS* in the Radiological Information System (RIS, Nexus.medRIS, v. 8.42, Nexus, Villingen-Schwenningen, Germany). Patient characterstics (age, gender), symptoms, date of symptom onset and RT-PCR results were extracted from electronic patient records.

### CT protocol

The patients underwent CT scans at two tertiary care hospitals. Chest CTs were performed on two different scanners (16 slice Somatom Sensation 16, 128-slice Definition FLASH, Siemens Healthcare, Forchheim, Germany). All CT acquisitions were obtained in supine position during end-inspiration. Intravenous contrast material was administered at the discretion of the radiologist considering the individual study indication. Automatic tube voltage selection was applied with a reference tube voltage of 120 kV. Tube current was regulated by an automatic tube current modulation technique with the reference mAs being 40–110. Collimation width was 0.625-0.75 mm. Axial planes were reconstructed with a slice thickness of 0.75–1.5 mm (96 CTs) and 3–4 mm (12 CTs) in lung kernel and with a slice thickness of 1–5 mm in soft tissue kernel. Additional sagittal and coronal MPRs were reconstructed with a slice thickness of 1–3 mm using lung and soft tissue kernel. The pictures were sent to a picture archiving and communication system (PACS, Syngo Imaging, Siemens, Erlangen, Germany).

### Image analysis

Two junior radiologists with subspeciality training in thoracic radiology evaluated each half of the CT studies on a PACS workstation. The evaluated patterns had been part of the training of the radiologists. In equivoval cases, a senior thoracic radiologist was consulted. The radiologists were blinded to clinical data, laboratory data and patient status. The Fleischner Society definition of CT features were applied when appropriate. The following parameters were analyzed:

- Ground-glass opacities (GGOs): hazy increased opacity of lung, with preservation of bronchial and vascular margins.
- Consolidation: homogeneous increase in pulmonary parenchymal attenuation that obscures the margins of vessels and airway walls.
- Crazy-paving pattern: thickened interlobular septa and intralobular lines superimposed on a background of GGO.
- Cavity: gas-filled space within consolidation.
- Bronchial dilatation: dilated (with respect to the accompanying pulmonary artery) non-tapering bronchus. The term ”dilatation“ instead of ”-ectasis“ was intentionally used in order to express that the pathology might be reversible.
- Vessel dilatation: diameter of vessel within or near opacifications clearly larger compared to vessels of the same generation in healthy lung tissue.
- Shape of opacification:
  - round
  - curvilinear/band-like
  - geographic (=opacification outlines the shape of multiple adjacent pulmonary lobules, sharply marginated).
- Margin of opacification:
  - unsharp
  - at least to some extent sharp.
- Lung lobes affected.
- Distribution of opacifications in the axial plane: predominantly peripheral, predominantly central, predominantly anterior, predominantly posterior, diffuse.
- Lymphadenopathy: diameter >10 mm in short axis.
- Subjective estimation of extent of parenchymal opacification: 0–33%: mild, 34–66%: moderate, 67–100%: severe.

### Statistical analysis

Age is presented as mean (standard deviation) and all categorical variables as absolute and relative frequencies. Descriptive values were calculated using *R*, v. 3.6.1 (The R Foundation for Statistical Computing Vienna, Austria.).

## Results

### Patient population and clinical symptoms

The study population consisted of 108 patients (male: 64 (59%)) with a mean age of 59.6 (SD 13.8). No CT had to be excluded, thus 108 CTs were evaluated. Clinical symptoms were fever in 74 patients (69%), cough in 86 patients (80%), dyspnea in 63 patients (58%) and fatigue in 52 patients (48%). Less frequently seen were gastrointestinal complaints (*n* = 22; 20%) and taste dysfunction (*n* = 20; 19%). The time interval between symptom onset and CT was mean 14.9 days (min 1 d, max 40 d, SD 37.3). For 70 patients, CT was performed within the first week after symptom onset. The time interval between CT and PCR was 2.3 days (SD 3.8). The results are presented in Table 1.

**Table 1.** Patient characteristics (*n* = 108)

­	n	%
**Mean age**	59,6 (SD 13.8)	
**Male**	64	59
**Fever (>37.5°C)**	74	69
**Cough**	86	80
**Dyspnea**	63	58
**Fatigue**	52	48
**Gastrointestinal complaints**	22	20
**Dysfunction of taste**	20	19

SD, standard deviation.

a Occasional missing values were counted as patients without symptoms.

### CT morphology

**Figure 1.**
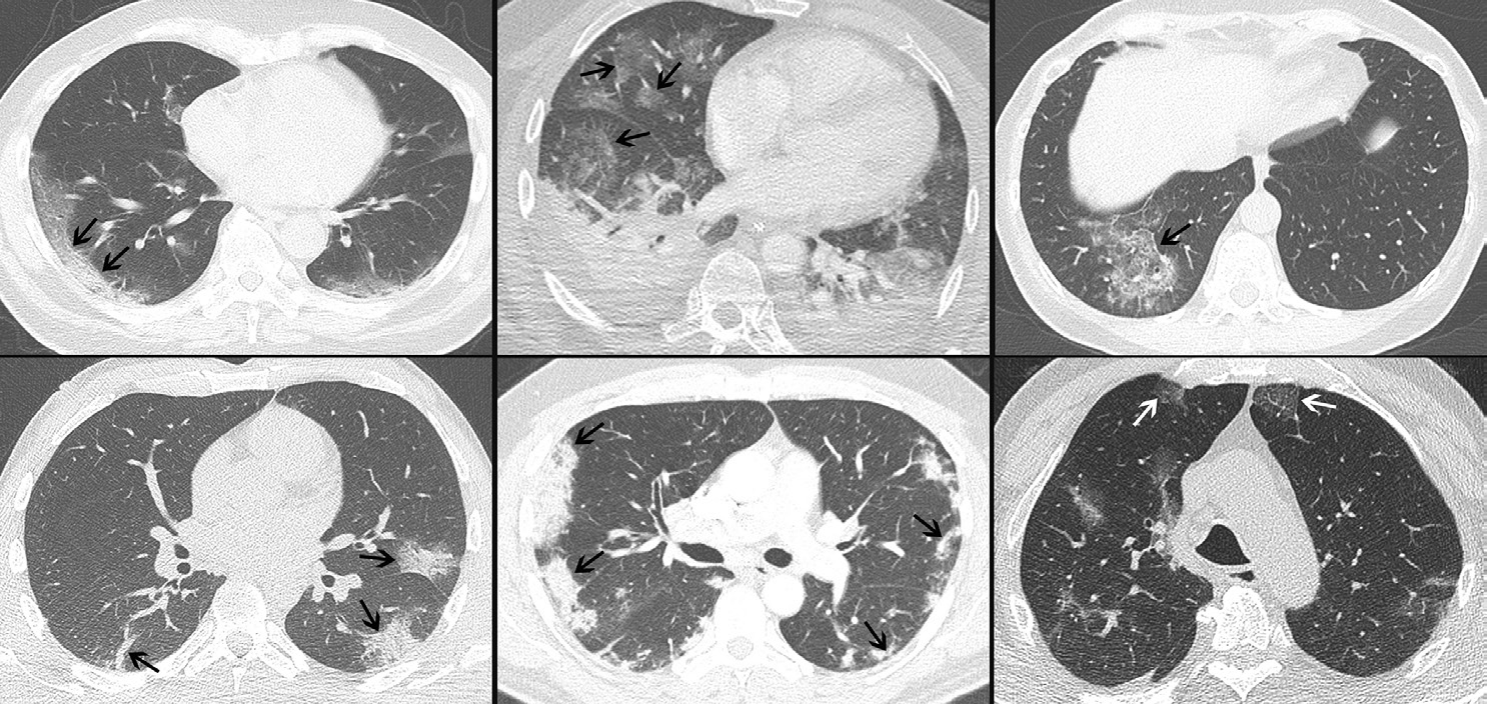
Axial MPRs of CT scans (1–4 mm slice thickness) of six patients with RT-PCR proven COVID-19 pneumonia. Opacities are at least to some extent sharply marginated with respect to the surrounding healthy lung tissue (arrows). This sign was found in 79% (*n* = 85) of the study population. MPR, multiplanar reconstruction; RT-PCR, reverse transcription polymerase chain reaction.

**Figure 2.**
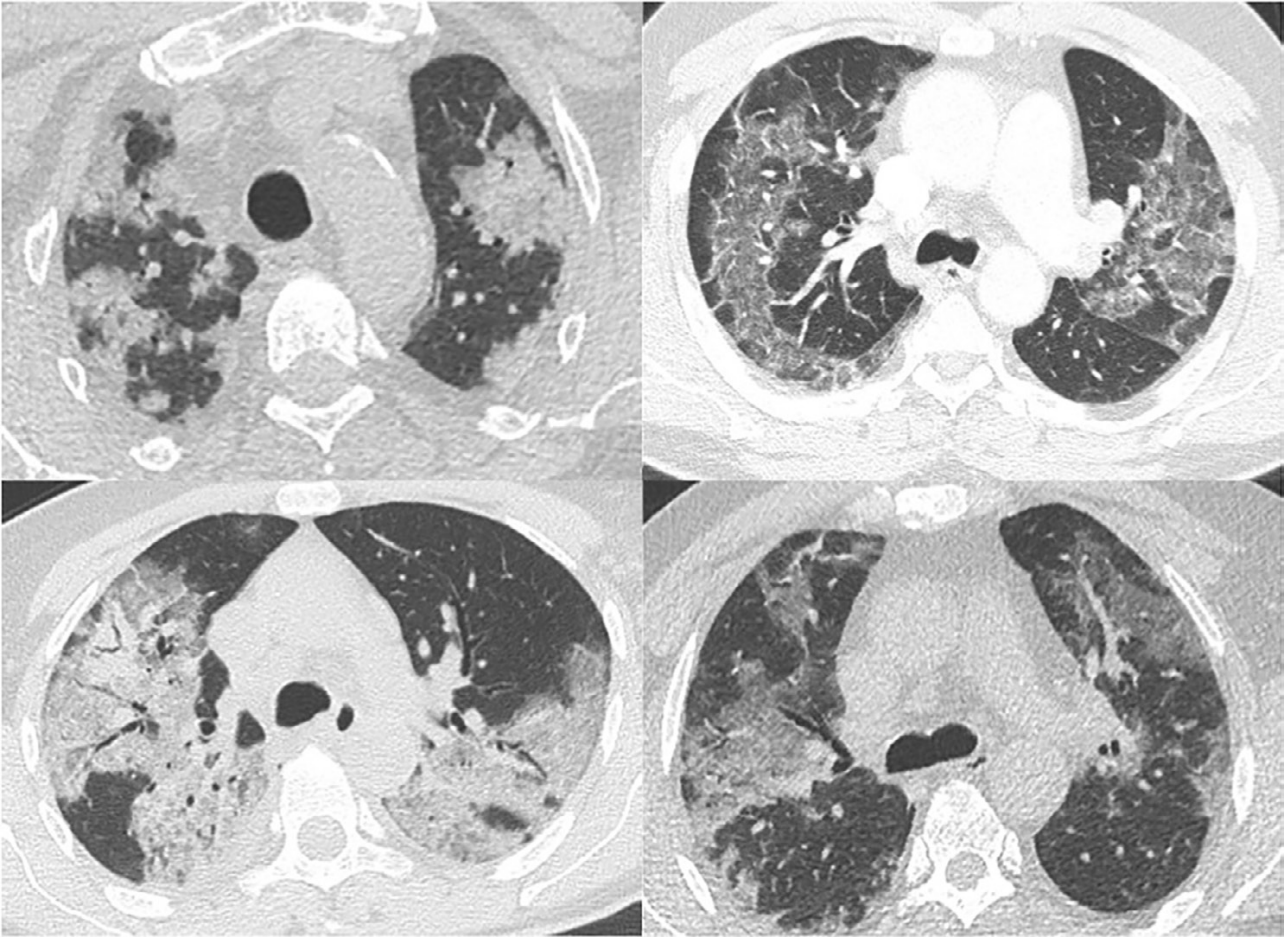
Axial MPRs of CT scans (1–4 mm slice thickness) of four patients with RT-PCR proven COVID-19 pneumonia. Opacities have a geographic shape (sharply marginated and outlining the contour of multiple adjacent pulmonary lobules). This sign was present in 27% (*n* = 29) of our study population. MPR, multiplanar reconstruction; RT-PCR, reverse transcription polymerase chain reaction.

GGO was the most frequent CT feature occuring in almost all patients (*n* = 104; 96%). The second most frequent CT feature was consolidation which was seen in 81 patients (75%). The crazy paving pattern was present in 18 patients (17%). Bronchial dilatation was seen in 23 (21%) cases and an air bronchogram in 31 (29%) cases. Vessel enlargement was observed in 51 patients (47%). Curvilinear or band-like configuration of densities occured in 47 patients (44%). A round shape of the opacification was detectable in 44 patients (41%). An at least to some extent sharp margin of the pulmonary lesions was present in 85 patients (79%) (Figure 1) and a geographic shape of opacifications in 29 patients (27%) (Figure 2). Lymphadenopathy was detected in 35 patients (32%). Pleural effusions were present in a minority of patients only (*n* = 17, 16%). Cavitation was not seen on any CT. The lung was almost always bilaterally affected (*n* = 101; 94%). The opacifications were predominantly located in the periphery of the lung (*n* = 75, 69%) and posterior lung segments (*n* = 85, 79%). The lower lobes were most often affected (right lower lobe: *n* = 104, 96%, left lower lobe: *n* = 90, 83%). The results are summarized in Tables 2 and 3.

**Table 2.** Frequency of CT features

CT feature	n	%
**Ground glass opacity**	104	96
**Consolidation**	81	75
**Crazy paving**	18	17
**Round shape of opacification**	44	41
**Sharp margin of opacification**	85	79
**Geographic shape of opacification**	29	27
**Curvilinear/bandlike opacification**	47	44
**Bronchial dilatation**	23	21
**Air bronchogram**	31	29
**Cavitation**	0	0
**Vessel enlargement**	51	47
**Lymphadenopathy**	35	32
**Pleural effusion**	17	16

**Table 3.** Distribution of opacifications within the lungs

Distribution	n	%
**Unilateral**	7	6
**Bilateral**	101	94
**Right upper lobe**	94	87
**Right middle lobe**	91	84
**Right lower lobe**	104	96
**Left upper lobe**	90	83
**Left lower lobe**	101	94
**Predominantly peripheral**	75	69
**Predominantly central**	0	0
**Diffuse**	45	42
**Predominantly anterior**	2	2
**Predominantly posterior**	85	79

## Discussion

The standard of reference for the diagnosis of COVID-19 is RT-PCR. However, reported sensitivites of RT-PCR range between 50 and 100%^12^ depending on several factors like viral load at sample site, location of swab, sampling procedure, quality of the specimen, test quality, regional prevalence of COVID-19 as well as timing during the course of the disease.^13^ Moreover, it takes hours to days before RT-PCR results are available. Authorities in China used chest CT to fill this gap because sensitivity of chest CT is high and in some studies even better than RT-PCR.^14,15^ CT morphology of COVID-19 pneumonia seems to exhibit remarkably similar features in many patients and CT morphology can be suggestive for the diagnosis.^16^ A combination of bilateral GGO and consolidation located in the periphery of the posterior segments predominantly of the lower lobes seems to be characteristic for COVID-19 pneumonia.^1–3,9,16,17^ The results of our study are in accordance with literature data.

Although morphology of COVID-19 pneumonia has been extensively studied specificity of chest CT for COVID-19 pneumonia is dismal with reported numbers as low as 25%.^12^ Obviously, specificity of CT for any disease depends on uniqueness of CT signs (density, size, shape, margin of lesion) and distribution patterns. However, GGO and consolidation with air bronchogram is seen in countless lung diseases. Crazy paving pattern would help to limit the list of differential diagnoses but occurs in a minority of patients with COVID-19 pneumonia only. A round shape of the opacifications is helpful because it occurs in a substantial number of cases with COVID-19 pneumonia and does not allow for many more differential diagnoses in the clinical setting of pneumonia in immunocompetent patients. The impact of vessel enlargement within/around the opaifications on specificity of CT is difficult to rate because to our knowledge this sign has not been evaluated in differential diagnoses like for example bacterial pneumonia, sterile pneumonitis, mucinous adenocarcinoma or interstitial lung disease. We evaluated two more CT signs which might increase specificity of CT for COVID-19 pneumonia: In the presented cohort, a sharp margin of opacifications with respect to the surrounding healthy tissue was observed in a high proportion of patients (*n* = 85, 79%). This is a sign which can be appreciated easily and is not often seen in other entities. Especially in non-COVID pneumonia margins of opacifications are usually indistinct. A geographic shape of lesions was present in 29 patients (27%). This further shortens the list of differential diagnoses. Sharp margin and geographic shape of opacifications most often occur in (cryptogenic) organizing pneumonia and alveolar proteinosis as well as in some cases of diffuse alveolar damage, alveolar hemorrhage and pulmonary edema. These entities should be distinguishable from COVID-19 pneumonia when considering clinical and laboratory data.

Our study has limitations. All included patients were RT-PCR positive for SARS-CoV-2. Our research protocol did not allow an investigation of the sensitivity or specificity of CT relative to RT-PCR. Moreover, patient outcomes were not yet avaliable at the time of submitting this article. Another limitation is that it was not systematically evaluated how often the reported signs are seen in other forms of pneumonia. Also, we did not analyze systematically for pre-existing lung conditions. Correlation of pre-existing lung disease with morphology of COVID-19 pneumonia and outcome warrants further investigation.

## Conclusion

We present the data of chest CTs of 108 patients from Germany with PCR-proven COVID-19. CT features and their frequency in this cohort are in accordance with published data. We identified two novel CT signs of COVID-19 pneumonia which have so far not been systematically evaluated. A sharp border and geographic shape of opacifications were frequently observed and might be helpful to increase specificity of CT for COVID-19 pneumonia.

## Summary

Two newly described CT signs (sharp margin and geographic shape of opacifications) were frequently observed in COVID-19 pneumonia.

### Key results

1. The most prevalent CT signs in a cohort of 108 patients from Germany with RT-PCR proven COVID-19 were GGOs (96%), consolidation (75%), vessel enlargement within/around opacities (47%) and air bronchogram (29%).

2. The shape of the opacifications was curvilinear (44%), round (41%) or geographic (27%). The opacifications were at least partially sharply marginated in 79% of cases.

3. The newly described CT features “sharp margin” and “geographic shape” of opacifications were frequently observed in patients with COVID-19 pneumonia and might help to increase specificity of CT.
